# Glycan Node Analysis of Plasma-Derived Extracellular Vesicles

**DOI:** 10.3390/cells9091946

**Published:** 2020-08-22

**Authors:** Sierra A. Walker, Jesús S. Aguilar Díaz De león, Sara Busatto, Gregory A. Wurtz, Abba C. Zubair, Chad R. Borges, Joy Wolfram

**Affiliations:** 1 Department of Biochemistry and Molecular Biology, Department of Physiology and Biomedical Engineering, Department of Transplantation, Mayo Clinic, Jacksonville, FL 32224, USA; walker.sierra1@mayo.edu (S.A.W.); busatto.sara@mayo.edu (S.B.); 2 School of Molecular Sciences and Virginia G. Piper Center for Personalized Diagnostics, The Biodesign Institute at Arizona State University, Tempe, AZ 85287, USA; jersus.aguilar@asu.edu; 3 Department of Physics, University of North Florida, Jacksonville, FL 32224, USA; g.wurtz@unf.edu; 4 Department of Laboratory Medicine & Pathology, Mayo Clinic, Jacksonville, FL 32224, USA; zubair.abba@mayo.edu; 5 Department of Nanomedicine, Houston Methodist Research Institute, Houston, TX 77030, USA

**Keywords:** carbohydrates, exosomes, glycan node analysis, microvesicle, size exclusion chromatography

## Abstract

Blood plasma is a readily accessible source of extracellular vesicles (EVs), i.e., cell-secreted nanosized carriers that contain various biomolecules, including glycans. Previous studies have demonstrated that glycans play a major role in physiological and pathological processes, and certain plasma glycans have been associated with disease conditions. However, glycome studies have been limited by a lack of analytical techniques with the throughput capacity necessary to study hundreds of clinical samples. This study is the first to characterize the EV plasma glycome based on all major glycan classes. The results based on glycan node analysis revealed, as expected, that plasma-derived EVs have distinct glycan features from donor-matched whole plasma. Specifically, glycan nodes corresponding to those observed in chondroitin sulfate, dermatan sulfate, type I keratan sulfate, and type II keratan sulfate were enriched on EVs. The identification of specific differences in glycan features in plasma vs. plasma-derived EVs is relevant for understanding the physiological role of EVs and as a reference for future diagnostic studies. Additionally, the results indicate that EV glycan nodes do not substantially differ among a small set of healthy donors. These results lay the framework for the further evaluation of all EV glycan classes as diagnostic markers, therapeutic targets, and biologically active components in health and disease.

## 1. Introduction

Blood plasma is an easily accessible biological fluid that has contact with all tissues in the body. Plasma has been used for diagnostic [1,2] and therapeutic [3,4] purposes, ranging from life-saving to symptom-reducing, for more than 100 years. For example, current advancements continue to push the bounds of therapeutic plasma exchange, recently growing to include treatment for inflammation-based optic neuritis [5], cardiac neonatal lupus, and Hashimoto’s encephalopathy, to name a few [6]. Plasma is also an ideal biological fluid for detecting the byproducts of various diseases, as it circulates throughout the body. Moreover, blood draws are common practice and only partially invasive. Several successful plasma-based diagnostic assays are in clinical use, such as the detection of non-small cell lung cancer [7], hepatitis [8], and human immunodeficiency virus (HIV) [9]. Many other promising diagnostic strategies are currently in development, including the detection of phosphorylated tau18 in Alzheimer’s disease [10]. Additionally, plasma components can mediate disease progression, making them potential candidates for therapeutic targets. For example, recent studies have shown that primary tumors can secrete components into the plasma that induce pre-metastatic niche formation [11]. However, it remains unclear which components in the plasma provide the most useful therapies, biomarkers, and therapeutic targets, as this complex biological fluid has not been fully characterized, which limits our understanding of the relevant physiological mechanisms involved in health and disease.

**Figure 1:**
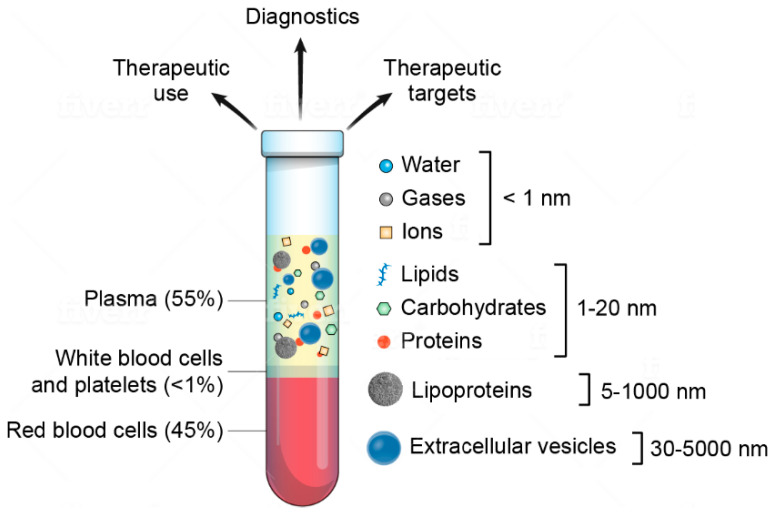
Schematic of blood content, highlighting plasma components. Components are not drawn to scale.

Major components of plasma include water, gases, ions, proteins, lipids, carbohydrates, lipoproteins, and extracellular vesicles (EVs) (Figure 1) [12,13,14]. EVs are nano-sized cell-secreted particles (30–5000 nm in diameter) that mediate intercellular communication through transfer of bioactive molecules involved in physiological and pathological processes [15,16,17,18,19]. Previously, several studies have characterized plasma EVs in disease, specifically focusing on the protein or microRNA content [20,21,22,23,24,25]. However, the plasma EV glycome has been mostly overlooked, primarily due to technical challenges in glycan analysis.

Glycobiology is an expanding field that has promising implications for improving the understanding of health and disease [26]. Previous studies on the plasma glycome have focused on the N-glycome as a promising diagnostic modality for longitudinal monitoring [27,28]. Additionally, studies have shown that certain plasma glycans (beyond N-linked glycans) are elevated in cancer patients when compared to healthy controls [29,30,31,32]. However, outside of a few studies targeting specific EV glycoproteins/proteoglycans or glycolipids [33,34,35], only a single study has assessed glycan expression levels in plasma based on association with EVs, and this analysis was limited to N-linked glycosylation [36]. The present study represents the first broad analysis of glycan expression (multiple glycan classes) in plasma-derived EVs. In this study, EVs are isolated from blood plasma through size-exclusion chromatography (SEC) columns, to remove small particles and lipoproteins, via column interactions with good yield and high purity. Understanding which glycans are associated with EVs is likely to improve diagnostic readouts and identification of novel therapeutic targets.

Differences in glycan expression in healthy donor-matched whole plasma and plasma-derived EVs were assessed by glycan node analysis (GNA), a medium-throughput method for analyzing carbohydrates. Introduced and analytically validated in detail in 2013 [37], GNA is a molecularly bottom-up gas chromatography-mass spectrometry (GC-MS)-based approach to glycan linkage analysis [37,38]. This method can be applied directly to complex biological matrices, covers all major classes of glycans, and condenses and captures unique glycan features, such as core fucosylation, α2-6-sialylation, bisecting N-acetylglucosamine (GlcNAc), and β1-6 branching, as single analytical signals, some of which serve as direct molecular surrogates for the activity of specific glycosyltransferases. Therefore, GNA is often capable of providing unique information that may remain undetected with conventional methods [30,31,32,37,38,39], and does so on a throughput scale, using instrumentation compatible with the needs of clinical research: a single analyst can reasonably prepare 60–75 samples a week for analysis [37]; and a single GC-MS instrument can easily analyze hundreds of samples a week.

To date, the GNA of whole blood plasma or serum from over 1000 of patients has proven effective at detecting and predicting progression, reoccurrence, and/or survival in lung cancer [30,32] and bladder cancer [31]. The implementation of an additional EV isolation step prior to GNA has the potential to improve the clinical sensitivity and specificity of plasma glycan nodes as cancer biomarkers, as tumor-derived EVs play a critical role in cancer formation and progression [40,41,42], and can be detected in the circulation [43]. In this study, the quantitative consistency of plasma-derived EV glycan nodes in healthy donors and quantitative differences in glycan nodes in whole plasma vs. donor-matched plasma-derived EVs are reported, including a discussion of the reasons for and potential clinical implications of these results.

## 2. Materials and Methods

### 2.1. Materials

Materials were acquired from the following sources: acetone from Avantor Performance Materials (Center Valley, PA, USA); methanol from Honeywell Burdick & Jackson (Muskegon, MI, USA); acetonitrile, methylene chloride, pierce spin columns (900 µL volume, Cat. No. 69705), GC-MS autosampler vials, mouse secondary antibody (Cat. No. 31450), high performance liquid chromatography (HPLC)-grade water, SuperSignal West-Femto maximum sensitivity substrate (enhanced chemiluminescence, ECL), Pierce bicinchoninic acid assay (BCA) Protein Assay Kit, Tween 80, 3-*N*-morpholinopropanesulfonic acid (MOPS) buffer, NuPAGE 12% bis-tris protein gels, Teflon-lined piercable caps, and cluster of differentiation 9 (CD9) antibody (Cat. No. 10626D) from Thermo Fisher Scientific (Waltham, MA, USA); dimethylsulfoxide (DMSO), iodomethane (99%, Cat. No. I8507), chloroform, trifluoroacetic acid (TFA), ammonium hydroxide, sodium borohydride, acetic anhydride, sodium acetate and sodium hydroxide beads (20-40 mesh, Cat. No. 367176) from Sigma-Aldrich (St. Louis, MO, USA); GC consumables from Agilent (Santa Clara, CA, USA); MS consumables from Waters (Milford, MA, USA); clinical grade sucrose buffer (5% sucrose, 50 mM Tris, and 2 mM MgCl, 08-735B) from Lonza (Bend, OR, USA); ultrapure sterile water from Rocky Mountain Biologicals (Missoula, MT, USA); calnexin antibody (Cat. No. 2433S) and rabbit secondary antibody (Cat. No. 7074S) from Cell Signaling Technology (Danvers, MA, USA); nitrocellulose transfer membrane from Abcam (Cambridge, MA, USA); HyClone phosphate buffered saline (PBS) from GE Healthcare (Chicago, IL, USA) Quantikine enzyme-linked immunosorbent assay (ELISA) for apolipoprotein B (apoB) (Cat. No. DAPD00) from R&D Systems (Minneapolis, MN, USA).

### 2.2. Methods

#### 2.2.1. Plasma Collection

The Mayo Clinic Biospecimens Review Group (ID: 17-010290) provided approval for the pre-clinical research use of human de-identified residual plasma. Plasma was collected in blood bags containing citrate phosphate dextrose-adenine-1 (CPCD-1), an anticoagulant, from healthy donors, for routine clinical use. Approximately 200 mL of plasma was frozen in transfusion bags at −20 °C, for up to one year before being considered expired. Plasma was thawed at 37 °C, stored at 4 °C, and if not used within five days of thawing, it was considered expired and repurposed for research use. Donor 1 and donor 3 were O+ blood type, while donor 2 was O-; all other donor demographics were unknown. Plasma samples were processed through SEC within one month of storage at −80 °C.

#### 2.2.2. EV Isolation

Izon qEV original 70 nm SeriesTM SEC columns were used to collect EV fractions from 500 µL of plasma according to the manufacturer’s instructions, including centrifugation at 1500× *g* for 10 min, to remove any remaining large particles after storage, followed by a final centrifugation step at 10,000× *g* for 10 min. These columns have a target particle size range of 70–1000 nm. Elution fractions were counted in 0.5 mL intervals, beginning after the void volume of 3 mL had passed through the column. Elution fractions seven through nine were pooled for analysis as ‘fraction 1′ (for a total of 1.5 mL) and elution fractions ten and eleven were pooled for analysis as ‘fraction 2′ (for a total of 1.0 mL). These two size exclusion fractions were isolated from each of three different blood plasma donors. For GNA, this process was repeated for each donor plasma sample in quadruplicate (four technical replicates for each fraction). GNA was performed on samples isolated in water (without cryoprotectant), as preservation of EV structure upon storage (−80 °C) prior to analysis is not required. For non-GNA based characterization, this process was repeated once for each donor in a previously published sucrose-based cryoprotectant (5% sucrose, 50 mM Tris, and 2 mM MgCl) [44], to preserve the structure of EVs upon storage in −80 °C, prior to analysis. GNA was performed in duplicate on all technical SEC replicates. GNA was performed six times on each whole plasma sample.

#### 2.2.3. Nanoparticle Tracking Analysis (NTA)

A NanoSight LM10 (Malvern Panalytical, Malvern, UK) was used to assess EV size distribution profiles and concentration. Fractions 1 and 2 were diluted 1:50 in HPLC grade water and three measurements were taken for a duration of one minute. Each replicate was measured under a continuous syringe pump flow rate of 40 µL/min. After adjusting the camera level to 12 and detection threshold to four for the first sample, only the focus was changed for the remaining samples, not the analytical software parameters.

#### 2.2.4. Atomic Force Microscopy (AFM) Imaging

AFM imaging was performed in tapping mode, using the dimension V Bioscope (Bruker, Billerika, MA, USA), equipped with PPP-FMR-SPL silicon tips (Nanosensors, Neuchatel, Switzerland). Briefly, EVs in sucrose buffer (50 µL) were diluted 1:20 in cell and molecular ultrapure sterile water. A fixed amount of properly diluted EV samples (50 μL) was then spin cast onto freshly cleaved mica sheets (grade V-1, thickness of 0.15 mm, size of 15 mm × 15 mm), using a model WS-650SZ-6NPP/LITE spin coater (Laurell, North Wales, PA, USA), at an acceleration of 600 rpm/s, up to a speed of 2000 rpm for 15 s. Imaging was performed using the Nanoscope Software 7.3 (Veeco, Plainview, NY, USA). Scan size ranged from 2.5 to 25 μm, and the scan speed ranged from 5 to 10 μm per second.

#### 2.2.5. Zeta Potential

The Zetasizer Nano ZS series ZEN 3600 (Malvern Panalytical, Malvern, UK) was used to determine the zeta potential, measured by laser Doppler micro-electrophoresis (Smoluchowski’s theory). Fractions 1 and 2 from all three donors were diluted 1:50 in PBS, for a final volume of 1 mL. Each dilution was placed into a plastic, disposable folded capillary cell, and any air bubbles were removed from the cuvette through gentle tapping prior to recording. Three sets of 20 measurements were taken for each sample.

#### 2.2.6. BCA

A BCA was performed to determine the protein concentration of each sample according to the manufacturer’s instructions.

#### 2.2.7. Western Blot

All samples were normalized to protein content. Sodium dodecyl-sulfate (6×) was added to a final concentration of 1×, and samples were boiled for five minutes at 90 °C. The equivalent of 1200 µg of protein was loaded in each well of a 12% polyacrylamide gel in MOPS buffer, and electrophoresis occurred for 1.5–2 h at 120 V. Transfer from the gel to a nitrocellulose membrane was completed at 200 mA for 1.5 h. The membrane was blocked at room temperature with 5% milk in TBS + 0.1% Tween (TBST) for two hours. Primary antibodies in 1% milk in TBST (1:500 dilution) were incubated overnight at 4 °C. After three ten-minute washes with TBST, secondary antibody in 1% milk in TBST (1:3000 dilution) was incubated for two hours at room temperature before three final ten minute washes with TBST and developing the membrane in an ECL solution for five minutes at room temperature in the dark. Images were collected using an Amersham 600 imager (GE Healthcare, Chicago, IL, USA).

#### 2.2.8. ApoB ELISA

Standards and samples were incubated with kit reagents, as per the manufacturer’s instructions. All samples were diluted to 2 × 10^7^ EVs per well and analyzed in triplicate. Best fit curve yielded an R2 value of 0.9989.

#### 2.2.9. GNA

The following glycan node analysis procedure was adapted from Borges et al.’s [37,38] EV samples suspended in water, which were concentrated by speed-vac to obtain final protein concentrations of 10–25 mg/mL, as measured by a BCA. Whole plasma samples were directly subjected to permethylation, following the addition of internal standard, as described below:

##### Permethylation, Nonreductive Release, and Purification of Glycans

Blood plasma or concentrated EV samples (10 µL) were added to 1.5 mL snap-cap polypropylene tubes, followed by the addition of DMSO (270 µL). Sodium hydroxide beads (~0.7 g) were collected in a ~1 mL Pierce spin column and washed with acetonitrile (ACN, 350 µL), followed by two rinses with DMSO (350 µL). The sample was combined with DMSO (270 µL) and iodomethane (105 μL), followed by immediate vortexing. The sample was then added to the pre-conditioned NaOH beads in the plugged microfuge spin column. The sample was allowed to sit in contact with the NaOH beads for 11 min, with occasional gentle stirring. The microfuge spin column was then unplugged and spun into a 2 mL sample collection tube for 30 s at 5000 rpm (1000 g in a fixed-angle rotor). The collected sample solution was quickly transferred into a silanized 13 × 100 mm glass test tube, containing 0.5 M NaCl in 0.2 M sodium phosphate buffer (pH 7) (3.5 mL). To maximize glycan recovery, the NaOH beads were washed twice with ACN (300 µL), with all spun-through liquid immediately transferred into the same silanized glass test tube. Liquid/liquid (L/L) extraction was then carried out by adding chloroform (1.2 mL) to each test tube, which was then capped and shaken well. After brief centrifugation to separate the layers, the upper aqueous layer was discarded and replaced with 0.5 M NaCl solution in 0.2 M sodium phosphate buffer (pH 7) (3.5 mL). After three L/L extraction rounds, the chloroform layer was recovered and dried under a gentle stream of nitrogen in a heater block set to 74 °C.

##### Hydrolysis, Reduction, and Acetylation

To perform acid hydrolysis, 2 M TFA (325 µL) was added to each sample, followed by heating at 121 °C for two hours. Samples were then dried under a gentle stream of nitrogen in a heater block set to 74 °C. To reduce the sugar aldehydes, freshly made 10 mg/mL sodium borohydride in 1 M ammonium hydroxide (475 µL) was added to dissolve each sample, followed by incubation at room temperature for one hour. To remove excess borate, methanol (MeOH, 63 µL) was added, mixed well and dried under nitrogen, followed by adding 9:1 (v/v) MeOH:acetic acid (125 µL), which was dried in like fashion. Samples were then fully dried in a vacuum desiccator for 20 min. To acetylate hydroxyl groups introduced by the acid hydrolysis step, deionized water (18 µL) was added to each test tube, to dissolve any precipitates. This was followed by the addition of acetic anhydride (250 µL) and sonication in a water bath for two minutes. Each sample was then incubated for ten minutes at 60 °C, followed by mixing with concentrated TFA (230 µL) and incubation again at 60 °C for ten minutes. To clean up the sample mixture prior to GC-MS, L/L extraction was performed twice, after adding dichloromethane (1.8 mL) and deionized water (2 mL) to each sample. The upper aqueous layer was discarded each round and the organic layer was then transferred to a silanized autosampler vial, and dried under nitrogen. Each sample was reconstituted in acetone (120 µL) and then capped in preparation for injection onto the GC-MS.

##### GC-MS

Samples were analyzed on an Agilent A7890 gas chromatograph equipped with a CTC PAL autosampler (Agilent Technologies, Santa Clara, CA, USA), coupled to a Waters GCT (time-of-flight: TOF) mass spectrometer (Milford, MA, USA). For each sample, 1 µL of the 120 µL total volume was injected at a split ratio of 20:1 onto a hot (280 °C), silanized glass liner (Cat. No. 5183-4647, Agilent Technologies, Santa Clara, CA, USA), containing a small plug of silanized glass wool. Volatilized sample components were separated on a 30-m DB-5ms GC column using helium as the carrier gas, at a constant flow rate of 0.8 mL/minute. The GC oven temperature was initially kept at 165 °C for 0.5 min, then increased to 265 °C at a rate of 10 °C/minute, followed by immediate ramping to 325 °C at a rate of 30 °C/minute, and finally held at 325 °C for 3 min. As sample components entered the mass spectrometer, they were subjected to electron ionization (70 eV, 250 °C). Positive-ion mode mass spectra from individual TOF pulses over a m/z range of 40–800 were summed every 0.1 s. The mass spectrometer was tuned and calibrated daily with perfluorotributylamine, to ensure reproducible relative abundances of electron ionization (EI) ions and mass accuracy within 10 ppm.

##### Data Processing

Summed extracted-ion chromatogram (XIC) peak areas for all glycan nodes were integrated with Quanlynx 4.1 software. A list of ions corresponding to each glycan node can be found elsewhere [37]. Peak areas were automatically integrated and manually verified, then exported to a spreadsheet for further analysis.

Individual hexoses were normalized to the sum of all endogenous hexoses, and individual N-acetylhexosamines (HexNAcs) were normalized to the sum of all endogenous HexNAcs. This approach provided relative glycan node profiling, and therefore direct comparison of glycan node profiles between EV and plasma samples. This does not facilitate quantitative comparisons between different glycan nodes within the same sample. Notably, terminal mannose is not chromatographically or mass spectrally resolved from terminal glucose (which is mostly derived from free blood glucose). If specific hexoses or HexNAcs were not reported as present, it was because their summed extraction ion chromatogram signal-to-noise ratio was less than ten.

## 3. Results

### 3.1. Physicochemical Characterization of Plasma-Derived EVs

**Figure 2:**
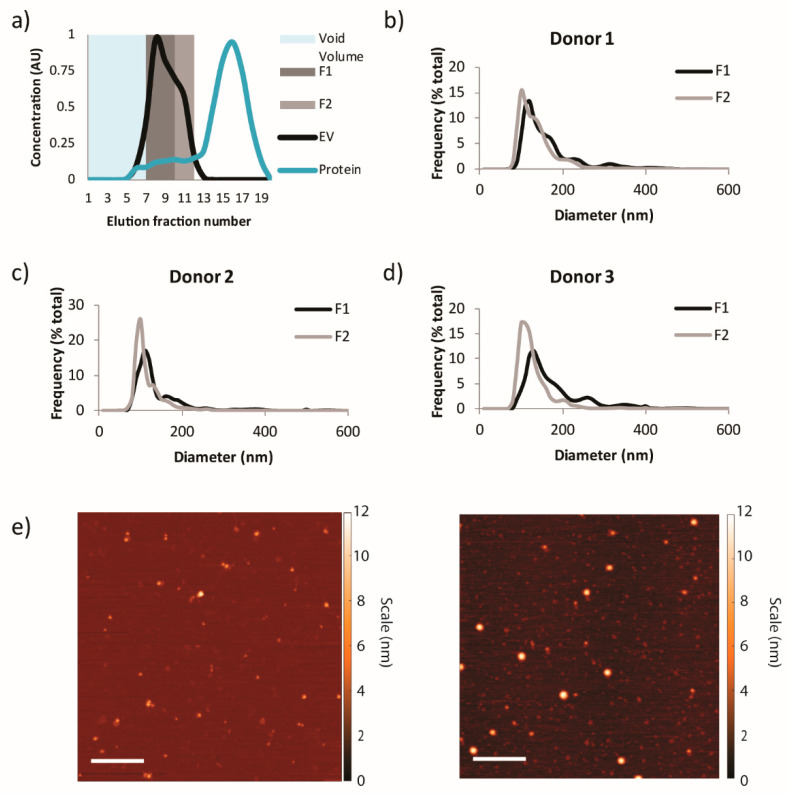
Size and morphology of plasma-derived extracellular vesicles (EVs) collected through size exclusion chromatography (SEC). (**a**) Schematic of expected content in SEC pooled elution fractions (F). F1 and F2 correspond to elution fractions 7–9 and 10–11, respectively. (**b**–**d**) Size distribution plots obtained from nanoparticle tracking analysis (NTA). (**e**) Representative atomic force microscopy images of fraction 1 (**left**) and fraction 2 (**right**) from donor 1. Scale bar, 500 nm.

**Figure 3:**
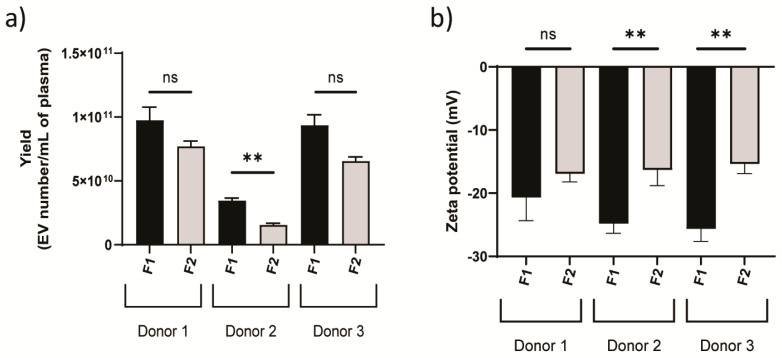
Yield and zeta potential of plasma-derived EVs. (**a**) Yield for each sample normalized to 1 mL of original plasma determined by NTA. (**b**) Zeta potential was obtained through laser Doppler micro-electrophoresis. Statistics by Student’s *t*-test. ** *p* < 0.0075; ns, not significant.

**Figure 4:**
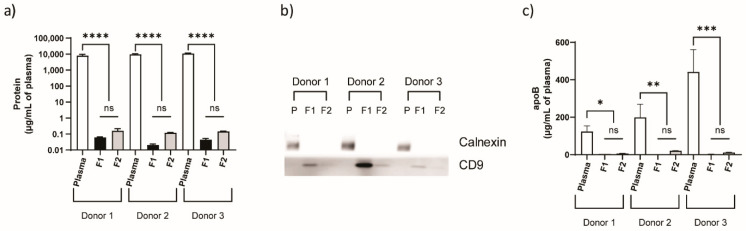
Protein markers of plasma-derived EVs. (**a**) Protein concentration for each sample normalized to 1 mL of original plasma. (**b**) Western blot of cluster of differentiation 9 (CD9) (EV marker) and calnexin (contaminant marker). P is plasma. (**c**) Enzyme linked immunosorbent assay (ELISA) for apolipoprotein B (apoB). Data represent mean ± s.d. (*n* = 3) Statistics by one-way analysis of variance (ANOVA). * *p* < 0.025; ** *p* < 0.004; *** *p* < 0.0007; ****, *p* < 0.0001; ns, not significant.

Isolation of intact EVs from biological samples containing many components can be performed with high purity using SEC, which outperforms ultracentrifugation (UC), the most commonly used method in the field [45]. A major source of impurities following conventional isolation procedures is lipoproteins, as these molecules have similar size range and density as EVs. Previously, it has been shown that over 90% of high-density lipoproteins (HDL) can be removed from hemoderivatives using SEC [46]. Moreover, apolipoprotein E (apoE), which is usually associated with chylomicrons, very-low-density lipoprotein (VLDL), and a subset of HDL particles in plasma [47], was previously shown to be absent from EV fractions isolated from lipoaspirate via SEC [48]. SEC has also been shown to reduce residual albumin, a contaminating protein, present in EV preparations in comparison to other isolation techniques [45,49]. In this study, healthy donor plasma was used as a source material for EVs, collected in fractions by SEC (Figure 2a). NTA (detectable range 10–1000 nm) showed populations of particles ranging from 80–400 nm, with the majority of particles being ~100 nm in size (Figure 2b–d). AFM revealed that EV fractions contained particles with a spherical shape with a similar size as observed by NTA (Figure 2e). The EV concentration was consistently higher in fraction 1 (elution fractions 7–9) compared to fraction 2 (elution fractions 10–11) (Figure 3a), and the former also displayed a more negative zeta potential (Figure 3b). Total protein was reduced by over 99% in all donor samples in the EV fractions obtained through SEC (Figure 4a). This accords with the minimal background detected by AFM (Figure 2e). EV fractions were shown to be negative for calnexin, a contaminant marker, and positive for CD9, a common EV marker (Figure 4b). Furthermore, apoB, typically associated with VLDL, intermediate-density lipoproteins (IDL) and low-density lipoprotein (LDL) [50], was dramatically reduced in the fractions compared to whole plasma (Figure 4c), indicating high purity in the isolated fractions.

### 3.2. GNA of Plasma-Derived EVs

**Figure 5:**
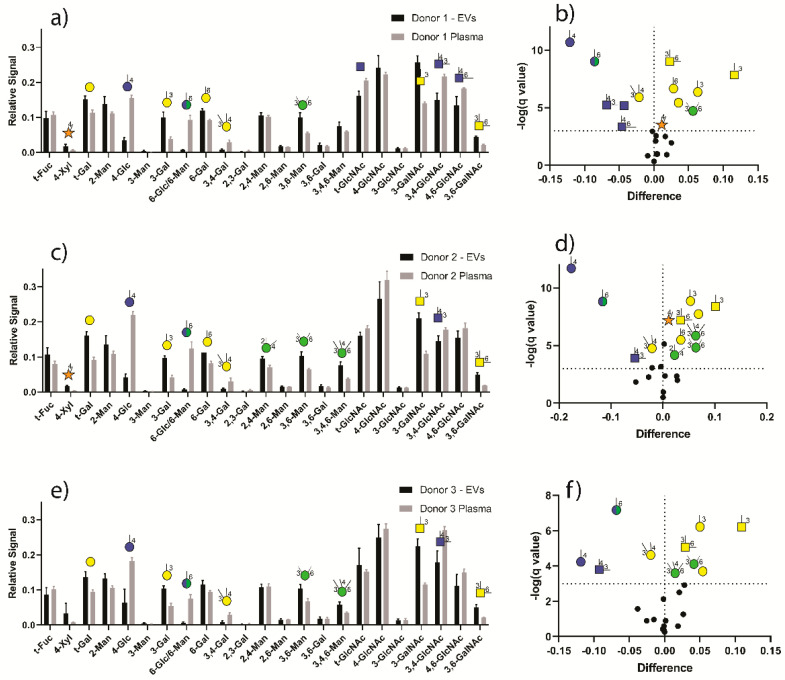
Comparison of EV vs. plasma glycan node profiles from three donors. Data represent extracted ion chromatogram (XIC) peak areas for each hexose or N-acetylhexosamine (HexNAc) within each sample that were normalized to the sum of all hexose or HexNAc XIC peak areas for that sample. Glycan nodes that were significantly different with regard to their relative abundance in EVs vs. plasma are summarized in the volcano plots (**b**,**d**,**f**) and depicted as their cartoon representations (defined by the x-axis in each bar graph (**a**,**c**,**e**)). For each glycan node, significant differences were determined by the Student’s t-test without assuming equal variance between groups. To correct for multiple comparisons, the false discovery rate was set at 0.1% according to the two-stage step-up procedure of Benjamini, Krieger and Yekutieli. Data represent mean ± s.d. (*n* = 8 for EVs and *n* = 6 for plasma).

There was a lack of statistically significant differences in GNA data from four SEC technical replicates from each donor, and data were statistically pooled to facilitate comparisons. As expected, numerous significant differences were observed between EV glycan node profiles (fraction 1 and fraction 2 combined) and those from plasma of the same donor (Figure 5). Moreover, 3-linked galactose (3-Gal), 3-linked N-acetylgalactosamine (3-GalNAc) and 3,6-linked N-acetylgalactosamine (3,6-GalNAc) residues were most strikingly and consistently elevated in EVs relative to whole plasma across all donors. Based on a comprehensive list of all known human glycosyltransferases, the reaction(s) they catalyze, and the glycan nodes that they produce (Table S2 of Borges et al. 2013 [37]), the most likely explanation for this phenomenon is that there is an increased relative abundance of proteoglycans (i.e., glycosaminoglycans/GAGs) in EVs, relative to whole plasma. In particular, these specific glycan nodes correspond to the unmodified hexoses and HexNAcs present in chondroitin sulfate and dermatan sulfate (3-GalNAc), as well as type II keratan sulfate (3-Gal and 3,6-GalNAc) [51]. Modified hexoses and HexNAc, such as those containing sulfate, phosphate or carboxyl groups, are not detected by the method [37]. This includes N-acetylneuraminic aid (NeuNAc) residues, which can only be detected indirectly as 6-linked galactose (6-Gal), or as a partial contributor to 3-linked galactose (3-Gal) [37]. Significantly elevated 4-linked xylose (4-Xyl) in EVs of two of the three donors accords well with this assignment, as this node serves as the reducing-end sugar attached directly to serine in chondroitin sulfate, dermatan sulfate and heparan sulfate GAGs, but is generally not observed in N-, O-, or lipid-linked glycans. Likewise, the enrichment of branched mannose residues in EVs is consistent with increased type I keratan sulfate.

Compared to plasma EVs, whole plasma was relatively enriched in 4-linked glucose (4-Glc), 6-linked glucose or mannose (which were indistinguishable by GC-MS; 6-Glc/6-Man), 3,4-linked N-acetylglucosamine (3,4-GlcNAc), and 3,4-linked galactose (3,4-Gal). The most likely sources of 4-Glc and 6-Glc in plasma are lactose (4-Glc), glycogen fragments (4- and 6-Glc) glycosylphosphatidylinositol (GPI)-anchored proteins. Furthermore, 3,4-GlcNAc represents antennary fucosylation and 3,4-Gal is most commonly found in glycolipids [37].

**Figure 6:**
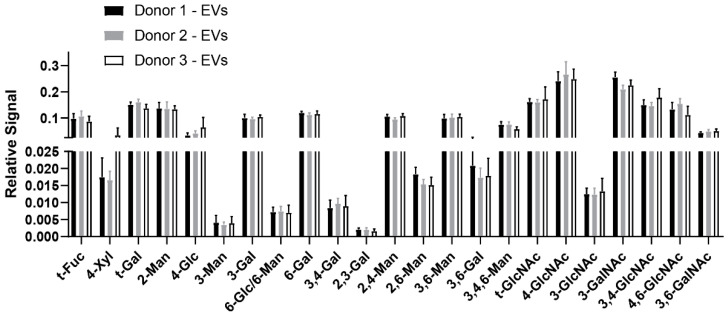
Comparison of EV glycan node expression across donors. Data represent XIC peak areas for each hexose or HexNAc within each sample, that were normalized to the sum of all hexose or HexNAc XIC peak areas for that sample. For each glycan node, differences between all pairwise combinations of donors were searched for with t-tests, using the two-stage linear step-up procedure of Benjamini, Krieger, and Yekutieli, with Q = 0.1% to correct for false discoveries. No significant differences were observed. Data represent mean ± s.d. (*n* = 8).

**Figure 7:**
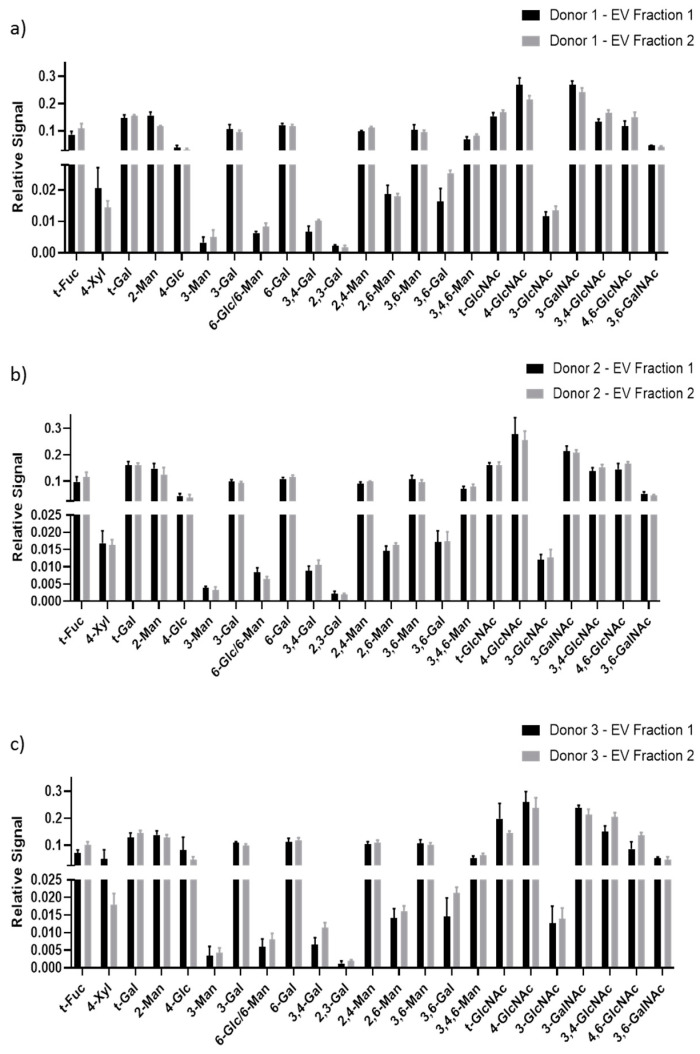
Comparison of EV fraction 1 (pooled elution fractions 7-9) and fraction 2 (pooled elution fractions 10–11) from each donor. Comparison of EV glycan nodes between SEC fractions 1 and 2 for each of three different donors (**a**–**c**). Data represent XIC peak areas for each hexose or HexNAc within each sample, that were normalized to the sum of all hexose or HexNAc XIC peak areas for that sample. For each glycan node, differences between fractions were searched for with a t-test, using the two-stage linear step-up procedure of Benjamini, Krieger, and Yekutieli, with Q = 0.1% to correct for false discoveries. No significant differences were observed. Data represent mean ± s.d. (*n* = 4).

A lack of statistically significant differences was observed between healthy donor EV glycans (Figure 6), indicating that the EV glycome profile may be consistent across healthy donor plasma. Similarly, a lack of statistically significant differences was observed between EV fractions 1 and 2, which held true across all three healthy plasma donors (Figure 7), demonstrating that these SEC-EV fractions can be pooled for glycan expression analysis (which was done in Figure 5 and Figure 6).

## 4. Discussion

The findings from this study confirm the expectation that glycan profiles of plasma-derived EVs are distinct from donor-matched whole plasma. The results also reveal that EV glycans do not substantially differ among a small set of healthy donors. Moreover, this study demonstrates that plasma EV fractions from SEC can be pooled for analysis, as glycan expression does not significantly differ among fractions. The results also reveal that glycan nodes corresponding to those observed in the GAG residues of proteoglycans such as chondroitin sulfate, dermatan sulfate, type I keratan sulfate, or type II keratan sulfate are relatively enriched on healthy plasma EVs. Chondroitin sulfate-decorated serglycin has previously been observed in serum-derived EVs, and found to play an important role in tumor-derived EV protein cargo loading [34]. Heparan sulfate and chondroitin sulfate-bearing syndecan-1 has been identified as a potential plasma EV-based marker of glioma [35]. Future studies, however, are necessary to fully elucidate the potential biological roles of the aforementioned biomolecules in this context. Notably, most approaches to glycan analysis avoid GAGs, and therefore would have been blind to the major findings reported here. Moreover, while GNA has the disadvantage of not being able to detect unique intact glycan structures, this prospect is generally irrelevant to GAG analysis.

It is worth noting that the glycan features reported in this study may include those present in the EV protein corona (biomolecular surface absorption). Currently, the EV protein corona remains a largely unexplored area of research [16,17,52,53]. Techniques developed for the study of the protein corona of synthetic nanoparticles are difficult to adapt to EV analysis, as such protocols would cause the damage or removal of EV biomolecules that are part of the membrane structure. In the case of synthetic nanoparticles, the protein corona has important implications for particle structure and function, including size, charge, aggregation, toxicity, immunological recognition, molecular targeting, biodistribution, uptake, and biocompatibility [17,54]. Future studies are necessary to adequately describe the binding modalities between identified glycan features and plasma-derived EVs, and the potential role of biomolecular absorption. It is possible that the EV protein corona could impact biomarker identification, and it is unclear whether the removal of the protein corona prior to analysis would impair or improve diagnostic capabilities. For example, it is possible that EVs absorb biomolecules from the tumor microenvironment prior to entering the circulation, which could contribute to identification of cancer-specific biomolecules.

Overall, identification of specific differences in glycan features in plasma vs. plasma-derived EVs is relevant for understanding the physiological role of EVs and as a reference for future diagnostic studies. The GNA of EVs is likely to be an important, cost effective, and time-efficient future method to assess glycan-dependent health from a simple blood draw, as EVs have been associated with multiple disease conditions [11,13,20,25,55]. Additionally, GNA of plasma EVs coupled with follow-up targeted studies could provide further understanding of various disease conditions and identification of potential therapeutic targets. GNA of plasma EVs may also aid in understanding therapeutic mechanisms involved in plasma-based therapies. This study represents the first broad analysis of glycan expression in plasma-derived EVs (based on all major glycan classes, not just N-glycans), and future studies with more samples will be necessary to further assess potential scientific and medical applications.
